# Increased Rates of Prolonged Length of Stay, Readmissions, and Discharge to Care Facilities among Postoperative Patients with Disseminated Malignancy: Implications for Clinical Practice

**DOI:** 10.1371/journal.pone.0165315

**Published:** 2016-10-25

**Authors:** Sarah B. Bateni, Frederick J. Meyers, Richard J. Bold, Robert J. Canter

**Affiliations:** 1 Division of Surgical Oncology, Department of Surgery, University of California, Davis Medical Center, Sacramento, California, United States of America; 2 Hematology/Oncology, Department of Internal Medicine, University of California, Davis Medical Center, Sacramento, California, United States of America; Virginia Mason Medical Center, UNITED STATES

## Abstract

**Background:**

The impact of surgery on end of life care for patients with disseminated malignancy (DMa) is incompletely characterized. The purpose of this study was to evaluate postoperative outcomes impacting quality of care among DMa patients, specifically prolonged length of hospital stay, readmission, and disposition.

**Methods:**

The American College of Surgeons National Surgical Quality Improvement Program (ACS-NSQIP) database was queried for years 2011–2012. DMa patients were matched to non-DMa patients with comparable clinical characteristics and operation types. Primary hepatic operations were excluded, leaving a final cohort of 17,972 DMa patients. The primary outcomes were analyzed using multivariate Cox regression models.

**Results:**

DMa patients represented 2.1% of all ACS-NSQIP procedures during the study period. The most frequent operations were bowel resections (25.3%). Compared to non-DMa matched controls, DMa patients had higher rates of postoperative overall morbidity (24.4% vs. 18.7%, p<0.001), serious morbidity (14.9% vs. 12.0%, p<0.001), mortality (7.6% vs. 2.5%, p<0.001), prolonged length of stay (32.2% vs. 19.8%, p<0.001), readmission (15.7% vs. 9.6%, p<0.001), and discharges to facilities (16.2% vs. 12.9%, p<0.001). Subgroup analyses of patients by procedure type showed similar results. Importantly, DMa patients who did not experience any postoperative complication experienced significantly higher rates of prolonged length of stay (23.0% vs. 11.8%, p<0.001), readmissions (10.0% vs. 5.2%, p<0.001), discharges to a facility (13.2% vs. 9.5%, p<0.001), and 30-day mortality (4.7% vs. 0.8%, p<0.001) compared to matched non-DMa patients.

**Conclusion:**

Surgical interventions among DMa patients are associated with poorer postoperative outcomes including greater postoperative complications, prolonged length of hospital stay, readmissions, disposition to facilities, and death compared to non-DMa patients. These data reinforce the importance of clarifying goals of care for DMa patients, especially when acute changes in health status potentially requiring surgery occur.

## Introduction

Although patients and clinicians consider oncologic outcome and survival the pre-eminent goals of cancer therapy, quality of life and avoidance of therapeutic morbidity, particularly among patients with stage IV cancer, are receiving increasing attention as important goals of care.[1–5] Prolonged length of hospital stay, intensive care unit stays, emergency room visits, hospital readmissions, and aggressive therapies, such as chemotherapy and surgery, have come under scrutiny given the increasing emphasis on improved palliative care and quality of life for patients near their end of life.[1–5] These issues create a dilemma for many surgeons, as patients with stage IV cancer commonly present with acute surgical conditions, such as bowel obstructions.[6, 7] In addition, surgeons are frequently faced with questions of whether surgical interventions should be performed electively on this patient population, since symptom palliation and prolongation of life are often potential benefits of surgery.[8, 9] However, we and others have shown that surgical intervention in this population is associated with high 30-day morbidity and mortality, with rates ranging from 27–44% and 9–11% respectively.[8–11] It is unclear to what extent this elevated post-surgical morbidity impacts other metrics of surgical outcomes among patients with disseminated malignancy (DMa) such as prolonged postoperative length of hospital stay, hospital readmission following the index surgical procedure, and disposition to facilities such as nursing homes.

The purpose of the present study was therefore to evaluate these outcomes, specifically prolonged length of hospital stay, readmissions, and disposition to facilities other than home, among DMa patients undergoing surgery since these outcomes can clearly impact the quality of life and overall trajectory of disease in patients with incurable malignancy. We hypothesized DMa patients would have substantially increased rates of these primary endpoints compared to non-DMa patients.

## Methods

### Data source, study population, and variable definitions

Data were obtained from the American College of Surgeons National Surgical Quality Improvement Program (ACS-NSQIP) from the years 2011 to 2012 (N = 986,034). ACS-NSQIP data collection methods have been described previously and have been shown to high data integrity and reliability.[12] These years were specifically selected due to the addition of data variables for disposition destination and 30-day hospital readmission. Analysis of readmission data was restricted to 2011 due to >99% data missing for 2012.

DMa patients undergoing surgical intervention were identified as described previously (N = 20,638 before exclusion).[11] ACS-NSQIP defines DMa as “patients who have cancer that: (1) has spread to one or more sites in addition to the primary site and (2) in whom the presence of multiple metastases indicates the cancer is widespread, fulminant or near terminal.”[13] Patients undergoing primary hepatic operation (N = 2,543) were excluded, as prior research has shown that hepatic operations are potentially curative in selected patients.[14–16]

We performed 1:1 matching of DMa to non-DMa patients who underwent an operation during this same time period. Patients were matched on the following characteristics: age, preoperative functional status, preoperative sepsis, procedure classification, and emergency procedure. These characteristics were specifically chosen as they have previously been shown to be independent predictors of postoperative morbidity and mortality for DMa patients.[10, 11] Standard ACS-NSQIP definitions were used to define these variables.[13] After matching, 123 DMa patients were excluded due to missing data from ≥1 of the matched variables, with a final cohort of 17,972 matched pairs.

Current procedural terminology (CPT) codes were used to classify procedures into the following categories: abdominal operations, neurosurgery, orthopedic, thoracic, urologic, gynecologic, skin/soft tissue, biopsy/lymph node excision, vascular, thyroid/parathyroid, otolaryngology, and cardiac operations. Abdominal operations were further categorized into small and large bowel resections, other small bowel and colorectal surgery, celiotomy and lysis of adhesions, pancreatic surgery, gastrectomy, other gastric surgeries, cholecystectomy, appendectomy, splenectomy, adrenal, biliary and other abdominal surgeries (e.g. excision of retroperitoneal mass). These categories were used for matching procedure type.

We then abstracted data on 3 demographic, 19 preoperative, 3 intraoperative, and 24 postoperative variables for DMa patients and matched controls. Standard ACS-NSQIP definitions were used for these variables with the following exceptions.[13] Multivisceral resections were identified based on the classification of the primary procedure CPT codes with additional procedure CPT codes. Postoperative prolonged length of hospital stay was defined as a length of stay ≥ the 75^th^ percentile for all operations for DMa patients and non-DMa matched controls, which was ≥10 days. This definition was consistent with prior published research using ACS-NSQIP.[17–19] Disposition to a facility other than home was defined as patient discharge to skilled care (e.g. transitional care, subacute hospital, ventilator bed, skilled nursing), unskilled facility (e.g. nursing home or assisted facility), other facility (e.g. chronic care, unskilled facility or assisted living), acute care or rehab facility.

Postoperative overall morbidity was defined as experiencing one or more of the following events within 30 days of the principal operation: superficial or deep wound infection, organ space infection, fascial dehiscence, pneumonia, reintubation, prolonged intubation, pulmonary embolism, progressive renal insufficiency, acute renal failure requiring dialysis, urinary tract infection, stroke, coma for >24 hours, peripheral nerve injury, cardiac arrest, myocardial infarction, graft/prosthesis/flap failure, deep vein thrombosis, reoperation, sepsis, and septic shock.[11]

Post-operative serious morbidity was defined as experiencing a complication associated with requiring further invasive procedures or leading to lasting disability, organ dysfunction, and/or death,[20] which included one or more of the following complications within 30 days of the principal operation: organ space infection, fascial dehiscence, pulmonary embolism, respiratory or cardiac failure requiring reintubation, prolonged intubation, acute renal failure requiring dialysis, reoperation, graft or flap failure requiring further procedures, stroke, coma, cardiac arrest or systemic shock.

Since all patient information was de-identified, this study was exempt from UC Davis Institutional Review Board approval.

### Statistical analysis

Differences in preoperative and intraoperative patient characteristics were compared between DMa and non-DMa patients using Chi-squared test for categorical variables and two-tailed independent t-tests for continuous variables. Logistic regression analysis was used to determine predictors of the primary and secondary outcomes, overall and serious morbidity, mortality, disposition to a facility, prolonged length of stay and hospital readmissions, for DMa patients. Multivariate conditional cox regression analysis was performed to determine risk of these primary and secondary outcomes for patients with DMa controlling for any residual confounding. Covariates included in the model comprised of gender, diagnoses of diabetes, hypertension, congestive heart failure (CHF), chronic obstructive pulmonary disease (COPD) or kidney disease requiring dialysis, symptoms of dyspnea, ascites, or weight loss, ventilator dependence, steroid use, albumin and multivisceral resection. DNR status and preoperative chemotherapy and radiotherapy were excluded from the multivariate analysis due to >55% of the data missing in the ACS-NSQIP database for these variables. Statistical significance was set at p<0.05. Case matching and all analyses were performed using SPSS software (IBM SPSS Statistics Version 22) and Statistical Analysis Software (SAS) version 9.4.

## Results

### General characteristics

From 2011 to 2012, 986,034 patients in the ACS-NSQIP database underwent surgical procedures with 2.1% (n = 20,638) diagnosed with DMa. After exclusion of patients who underwent a primary hepatic operation (n = 2,543) and who were not successfully matched (n = 123), 17,972 DMa patients were identified.

Preoperative and intraoperative patient characteristics of the matched pairs are depicted in **Table 1**. As expected, there were no significant differences between DMa patients and matched cohorts with respect to age, functional status, preoperative sepsis, and emergency operations. However, there were significant differences between the groups with respect to gender, DNR status, comorbid health conditions, BMI, preoperative weight loss, recent history of chemotherapy or radiation therapy, preoperative laboratory values, and rates of multivisceral resections.

**Table 1:** Preoperative and Intraoperative Characteristics for Disseminated Malignancy Patients and Matched Non-Disseminated Patients.

Demographics		Disseminated Malignancy Patients	Non-Disseminated Malignancy Patients	P Value
		N = 17,972	N = 17,972	
		N (% or ± SD)	N (% or ± SD)	
**Age**		62 (± 13)	62 (± 14)	NS*
**Female Gender**		9,484 (52.8%)	9,729 (54.3%)	P<0.01
***Ethnicity***				
	**Caucasian**	13,725 (76.4%)	13,965 (77.7%)	P<0.01
	**African American**	1,638 (9.1%)	1,660 (9.2%)	NS
	**Asian**	642 (3.6%)	392 (2.2%)	P<0.01
	**Native American/ Alaskan Native**	131 (0.7%)	174 (1.0%)	P<0.05
	**Hispanic**	871 (5.3%)	858 (5.3%)	NS
	**Unknown**	1,836 (10.2%)	1,781 (9.9%)	NS
**BMI**		27.2 (± 6.6)	28.7 (± 7.2)	P<0.01
**DNR**^+^		187 (2.6%)	62 (0.7%)	P <0.01
**Diabetes Mellitus**		2,669 (14.8%)	3,127 (17.4%)	P<0.01
**Hypertension**		8,649 (48.1%)	9,533 (53.0%)	P<0.01
**CHF**		168 (0.9%)	225 (1.3%)	P<0.01
**COPD**		1,343 (7.5%)	1,295 (7.2%)	NS
**Dyspnea**		2,372 (13.2%)	2,180 (12.1%)	P<0.01
**Ventilator Dependence**		204 (1.1%)	252 (1.4%)	P<0.05
**Ascites**		1,127 (6.3%)	186 (1.0%)	P<0.01
**Renal Failure Requiring Dialysis**		152 (0.8%)	432 (2.4%)	P<0.01
**Steroid Use**		1,810 (10.1%)	903 (5.0%)	P<0.01
**Weight Loss 6 months prior to Surgery**		1,998 (11.1%)	646 (3.6%)	P<0.01
**Chemotherapy < = 30 days**^+^		2,014 (27.9%)	252 (2.9%)	P<0.01
**Radiotherapy last 90 days**^+^		484 (6.8%)	118 (1.4%)	P<0.01
***Impaired Functional Status***		1,315 (7.3%)	1,315 (7.3%)	NS
	**Partially Dependent**	1,097 (6.1%)	1,097 (6.1%)	NS
	**Totally Dependent**	218 (1.2%)	218 (1.2%)	NS
**Preoperative Sepsis**		2,055 (11.4%)	2,055 (11.4%)	NS
***Preoperative Laboratory Value*s**				
	**Creatinine**	0.96 (± 0.67)	1.09 (± 1.02)	P<0.01
	**Albumin**	3.50 (± 0.78)	3.71 (± 0.76)	P<0.01
	**Hematocrit**	35.6 (± 5.8)	38.4 (± 5.7)	P<0.01
**Emergency Operation**		2,407 (13.4%)	2,407 (13.4%)	NS
**Multivisceral Resection**		1,930 (10.7%)	780 (4.3%)	P<0.05

* NS, Not Significant, p ≥ 0.05.

^+^ > 55% data missing from ACS-NSQIP database for 2011 and 2012.

As shown in **Table 1**, DMa patients had a slightly lower BMI (27.2 ±6.6 vs. 28.7 ±7.2, p<0.01) and were more likely to have had significant preoperative weight loss (11.1% vs. 3.6%, p<0.01), ascites (6.3% vs. 1.0%, p<0.01), preoperative systemic steroids for a chronic medical condition (10.1% vs. 5.0%, p<0.01), and preoperative chemotherapy and radiation therapy (27.9% vs. 2.9% and 6.8% vs. 1.4% respectively, p<0.01). DMa patients had a lower creatinine (0.96 ±0.67 vs. 1.09 ±1.02, p<0.01), albumin (3.5 ±0.78 vs. 3.71 ±0.76, p<0.01), and hematocrit (35.6 ±5.8 vs. 38.4 ±5.7, p<0.01) and were more likely to undergo a multivisceral resection (10.7% vs. 4.3%, p<0.01).

Non-DMa patients were more likely to have a comorbid health condition including diabetes mellitus (17.4% vs. 14.8%, p<0.01), hypertension (53.0% vs. 48.1%, p<0.01), congestive heart failure (CHF) (1.3% vs. 0.9%, p<0.01), and renal failure requiring dialysis (2.4% vs. 0.8%, p<0.01).

As depicted in **Table 2**, the majority of operations performed on DMa patients were abdominal operations (50.3%, n = 9,040) with bowel resections being the most common (25.3%, n = 4,538), followed by other small bowel and colorectal procedures (6.8%, n = 1,222), and celiotomy/lysis of adhesions (4.0%, n = 723). Neurosurgical operations were the second most common type of operation (10.5%, n = 1,881) followed by orthopedic surgery (7.1%, n = 1,275).

**Table 2:** Procedures Performed on Patients with Disseminated Malignancy.

Type of Procedure		N (%)
***Abdominal***		9,040 (50.3%)
	**Bowel Resection**	4,538 (25.3%)
	**Other Small Bowel/Colorectal Surgery**	1,222 (6.8%)
	**Celiotomy/Lysis of Adhesions**	723 (4.0%)
	**Pancreas**	384 (2.1%)
	**Other Gastric Surgery**	318 (1.8%)
	**Cholecystectomy**	320 (1.8%)
	**Hernia**	418 (2.3%)
	**Gastrectomy**	128 (0.7%)
	**Appendectomy**	112 (0.6%)
	**Splenectomy**	98 (0.6%)
	**Adrenal**	86 (0.5%)
	**Biliary**	83 (0.5%)
	**Other**	610 (3.4%)
**Neurosurgery**		1,881 (10.5%)
**Orthopedic**		1,275 (7.1%)
**Thoracic**		1,260 (7.0%)
**Urologic**		992 (5.5%)
**Gynecologic**		986 (5.5%)
**Skin/Soft Tissue**		920 (5.1%)
**Lymph Node/Biopsy**		771 (4.3%)
**Vascular**		395 (2.2%)
**Thyroid/Parathyroid**		198 (1.1%)
**Ear Nose Throat**		170 (0.9%)
**Cardiac**		81 (0.5%)
**Total**		17,972 (100%)

### Predictors of 30-day morbidity, mortality, prolonged length of stay, readmissions, and disposition to facilities other than home among disseminated malignancy patients

Multivariate logistic regression analysis showed common predictors of overall and serious morbidity and mortality included preoperative sepsis (OR = 1.42, 95%CI = 1.32–1.53, p<0.001 for overall morbidity; OR = 1.53, 95%CI = 1.41–1.65, p<0.001 for serious morbidity; OR = 1.41 (95%CI = 1.29–1.54 for mortality), emergency operations (OR = 1.51, 95%CI = 1.35–1.70, p<0.001 for overall morbidity; OR = 1.61, 95%CI = 1.42–1.84, p<0.001 for serious morbidity; OR = 1.81, 95%CI = 1.54–2.11, p<0.001 for mortality), lower albumin (OR = 0.68, 95%CI = 0.64–0.72, p<0.001 for overall morbidity; OR = 0.69, 95%CI = 0.65–0.74, p<0.001 for serious morbidity; OR = 0.43, 95%CI = 0.39–0.47 for mortality), and ventilator dependence (OR = 2.07, 95%CI = 1.46–2.92, p<0.001 for overall morbidity; OR = 2.90, 95%CI = 2.05–4.08, p<0.001 for serious morbidity; OR = 1.52, 95%CI = 1.05–2.18, p<0.05 for mortality). Impaired functional status was a predictor of overall morbidity (OR = 1.13, 95%CI = 1.01–1.27, p<0.05) and mortality (OR = 1.71, 95%CI = 1.49–1.96, p<0.001). Multivisceral resection and male gender were predictors of overall and serious morbidity (Multivisceral resection: OR = 2.32, 95%CI = 2.07–2.60, p<0.001 for overall morbidity and OR = 2.18, 95%CI = 1.91–2.49, p<0.001 for serious morbidity; Gender: OR = 0.87, 95%CI = 0.80–0.94, p<0.01 for overall morbidity and OR = 0.80, 95%CI = 0.73–0.89, p<0.001 for serious morbidity), but not mortality (p≥0.05). The remaining predictors of overall morbidity included diagnoses of COPD, CHF and hypertension and lower hematocrit (p<0.05). For serious morbidity other predictors included symptoms of dyspnea and diagnoses of COPD and kidney disease requiring dialysis (p<0.05). Older age, symptoms of dyspnea, ascites, and weight loss, preoperative steroid use, increased creatinine and lower hematocrit were additional predictors of mortality (p<0.05).

Predictors of discharge to a facility and prolonged length of stay included older age (OR = 1.04, 95%CI = 1.04–1.05, p<0.001 and OR = 1.004, 95%CI = 1.001–1.008, p<0.01 respectively), impaired functional status (OR = 2.11, 95%CI = 1.89–2.37, p<0.001 and OR = 1.34, 95%CI = 1.18–1.51, p<0.001), lower albumin (OR = 0.58 95%CI = 0.54–0.62, p<0.001 and OR = 0.42, 95%CI = 0.39–0.45, p<0.001), lower hematocrit (OR = 0.99, 95%CI = 0.98–0.99, p<0.01 and OR = 0.97, 95%CI = 0.96–0.98, p<0.001) and emergency operations (OR = 1.34, 95%CI = 1.18–1.52, p<0.001 and OR = 1.54, 95%CI = 1.36–1.74, p<0.001). Diagnoses of diabetes and hypertension and preoperative steroid use were also predictors of discharge to a facility among DMa patients, p<0.05. Male gender, African American ethnicity, preoperative diagnoses of COPD and CHF, sepsis, ascites, weight loss, and multivisceral resections were additional predictors of prolonged length of stay, p<0.05. Male gender, ascites and low albumin were the only statistically significant predictors of hospital readmission, p<0.05.

### Morbidity and mortality

DMa patients had significantly higher rates of postoperative overall 30-day morbidity (24.4% vs. 18.7%; aHR = 1.35, 95%CI = 1.27–1.44, p<0.001), serious morbidity (14.9% vs. 12.0%; aHR = 1.24, 95%CI = 1.16–1.34, p<0.001) and mortality (7.6% vs. 2.5%; aHR = 3.89, 95%CI = 3.36–4.49 p<0.001) compared to matched non-DMa patients (**Table 3**). Subgroup analysis of DMa patients who underwent a bowel resection (n = 4,538) showed similar results. As shown in **Table 4**, 36.8% (n = 1,669) of DMa patients experienced a postoperative complication after bowel resection compared to 29.2% (n = 1,324) of non-DMa patients (aHR = 1.35, 95%CI = 1.22–1.49, p<0.001), 21.9% (n = 993) of DMa patients experienced a serious complication compared to 18.2% (n = 825) of non-DMa patients (aHR = 1.22, 95%CI = 1.08–1.38, p<0.001), and 9.3% (n = 424) of DMa patients died within 30 days after the index operation compared to 4.1% (n = 186) of non-DMa patients (aHR = 2.99, 95%CI = 2.36–3.78, p<0.001).

**Table 3:** Postoperative Outcomes among Disseminated Malignancy Patients Compared to Matched Non-Disseminated Malignancy Patients.

Variable	Disseminated Malignancy Patients	Non-Disseminated Malignancy Patients	Adjusted Hazard Ratio	P Value
	N (%)	N (%)	(95% CI)	
Prolonged Length of Stay*	5,781 (32.2%)	3,554 (19.8%)	1.85 (1.74–1.97)	P<0.001
**Readmission within 30 days**	1,157 (15.7%)	1,293 (9.6%)	1.60 (1.42–1.79)	P<0.001
**Discharge to Facility**	2,915 (16.2%)	2,309 (12.9%)	1.36 (1.26–1.47)	P<0.001
**Overall 30-Day Morbidity**	4,393 (24.4%)	3,362 (18.7%)	1.35 (1.27–1.44)	P<0.001
**30-Day Serious Morbidity**	2,673 (14.9%)	2,159 (12.0%)	1.24 (1.16–1.34)	P<0.001
**30-Day Mortality**	1,361 (7.6%)	449 (2.5%)	3.89 (3.36–4.49)	P<0.001

*Length of Stay (LOS). Prolonged LOS defined as hospitalization LOS ≥75^th^ percentile.

**Table 4:** Sub-Group Analysis of Postoperative Outcomes among Disseminated Malignancy Patients after Bowel Resections and Celiotomy/Lysis of Adhesions.

Variable		Disseminated Malignancy Patients	Non-Disseminated Malignancy Patients	Adjusted Hazards Ratio	P Value
		N (%)	N (%)	(95% CI)	
**Bowel Resections**		**N = 4,538**	**N = 4,538**		
	Prolonged Length of Stay*	2,155 (47.5%)	1,431 (31.5%)	1.83 (1.65–2.03)	P<0.001
	**Readmission within 30 days**	279 (15.4%)	390 (12.3%)	NS^+^	P = 0.10
	**Discharge to Facility**	800 (17.6%)	637 (14.0%)	1.38 (1.20–1.57)	P<0.001
	**Overall 30-Day Morbidity**	1,669 (36.8%)	1,324 (29.2%)	1.35 (1.22–1.49)	P<0.001
	**30-Day Serious Morbidity**	993 (21.9%)	825 (18.2%)	1.22 (1.08–1.38)	P<0.01
	**30-Day Mortality**	424 (9.3%)	186 (4.1%)	2.99 (2.36–3.78)	P<0.001
**Celiotomy/Lysis of Adhesions**		**N = 723**	**N = 723**		
	Prolonged Length of Stay*	321 (44.4%)	260 (36.0%)	NS^+^	P = 0.06
	**Readmission within 30 days**	45 (13.5%)	54 (8.1%)	NS^+^	P = 0.17
	**Discharge to Facility**	102 (14.1%)	92 (12.7%)	NS^+^	P = 0.84
	**Overall 30-Day Morbidity**	169 (23.4%)	165 (22.8%)	NS^+^	P = 0.51
	**30-Day Serious Morbidity**	111 (15.4%)	113 (15.6%)	NS^+^	P = 0.39
	**30-Day Mortality**	118 (16.4%)	38 (5.3%)	6.27 (3.06–13.65)	P<0.001

* Length of Stay (LOS). Prolonged LOS defined as hospitalization LOS ≥75^th^ percentile.

^+^ NS, Not Significant, P ≥ 0.05.

DMa patients who underwent a celiotomy/lysis of adhesions did not have statistically significant differences with respect to morbidity compared to non-DMa patients, but did have a substantial increase in mortality (16.4% vs. 5.3%, aHR = 6.27, 95%CI = 3.06–13.85, p<0.001).

### Prolonged length of stay, readmission, and disposition

**Table 3** depicts rates of prolonged length of hospital stay, 30-day readmissions, and disposition to a facility for DMa patients who underwent any operation. Overall, DMa patients had significantly higher rates of prolonged length of hospital stays (32.2% vs. 19.8%, aHR = 1.85, 95%CI = 1.74–1.97, p<0.001), readmission (15.7% vs. 9.6%, aHR = 1.60, 95%CI = 1.42–1.79, p<0.001), and discharges to facility (16.2% vs. 12.9%, aHR = 1.36, 95%CI = 1.26–1.47, p<0.01) compared to non-DMa patients.

Subgroup analysis of bowel resections demonstrated similar results. As shown in **Table 4**, DMa patients who underwent bowel resections had significantly higher rates of prolonged length of stay (47.5% vs. 31.5%, aHR = 1.83, 95%CI = 1.65–2.03, p<0.001) and discharge to a facility (17.6% vs. 14.0%, aHR = 1.38, 95%CI = 1.20–1.57, p<0.001). However, for patients who underwent celiotomy/lysis of adhesions, although rates of these three primary endpoints were higher among DMa patients compared to non-DMa patients, these differences were not statistically significant in the multivariate analysis.

Importantly, as shown in **Table 5**, DMa patients who did not experience any ACS-NSQIP postoperative complication nevertheless experienced significantly higher rates of prolonged length of stay (23.0% vs. 11.8%, aHR = 2.12, 95%CI = 1.93–2.32, p<0.001), readmissions (10.0% vs. 5.2%, aHR = 1.75, 95%CI = 1.44–2.13, p<0.001), discharge to a facility (13.2% vs. 9.5%, aHR = 1.48, 95%CI = 1.32–1.65, p<0.001), and 30-day mortality (4.7% vs. 0.8%, aHR = 7.47, 95%CI = 5.38–10.39, p<0.001) compared to non-DMa patients.

**Table 5:** Postoperative Outcomes for Patients Who Did Not Experience Any Complication.

Variable	Disseminated Malignancy Patients	Non-Disseminated Malignancy Patients	Adjusted Hazards Ratio	P Value
	N = 13,577	N = 14,609		
	N (%)	N (%)	(95% CI)	
Prolonged Length of Stay*	3,125 (23.0%)	1,728 (11.8%)	2.12 (1.93–2.32)	P<0.001
**Readmission within 30 days**	546 (10.0%)	562 (5.2%)	1.75 (1.44–2.13)	P<0.001
**Discharge to Facility**	1,786 (13.2%)	1,386 (9.5%)	1.48 (1.32–1.65)	P<0.001
**30-Day Mortality**	641 (4.7%)	113 (0.8%)	7.47 (5.38–10.39)	P<0.001

*Length of Stay (LOS). Prolonged LOS defined as hospitalization LOS ≥75^th^ percentile.

## Discussion

Our findings that DMa patients were at greater risk of acute postoperative morbidity and mortality are consistent with prior research.[8–11] However, most importantly, we observed that DMa patients undergoing surgery had significantly higher rates of prolonged length of hospital stay, readmissions, and disposition to facilities other than home compared to non-DMa matched controls. These data highlight the dilemma that physicians and surgeons commonly face when presented with patients with DMa who are diagnosed with an acute surgical condition and/or condition that potentially may benefit from surgical palliation.

For example, surgeons are frequently consulted for malignant bowel obstruction, a diagnosis common in the setting of both ovarian and gastrointestinal malignancies, with rates as high as 28–51%.[6, 21] Although malignant bowel obstruction may be successfully managed by non-operative approaches, bowel obstruction is still considered a surgical condition, as in some cases the consequences of a delay in indicated surgical treatment may be severe.[21–24] However, our results emphasize not only the significant risks of acute morbidity and mortality associated with performing surgery, which others have also observed,[10, 11, 22, 23] but also the increased risks of prolonged hospital stays, hospital readmissions, and discharge to facilities. For example, in our analysis, DMa patients who underwent bowel resections, a common surgery performed for obstruction, were more likely to experience prolonged length of stay and disposition to a facility other than home. In addition, these patients with DMa were more than twice as likely to die within 30 days of the surgery compared to patients without DMa. This increased risk of mortality was also present for DMa patients who underwent celiotomy or lysis of adhesions as well, operations also commonly performed for bowel obstruction.

These findings complement previous research comparing medical versus surgical outcomes for patients hospitalized with malignant bowel obstruction. Henry et al. observed in this retrospective single institution study that patients presenting with malignant bowel obstruction who underwent surgical intervention experienced longer hospital stays and were more likely to be discharged to an extended care facility compared to patients who were treated with medical management.[22]

Data such as these highlight the need for physicians and surgeons to engage in goals of care and end of life discussions with their patients prior to pursuing surgical intervention on patients with DMa. Patients should be provided with an accurate assessment of the potential risks of surgery, including incidence of prolonged length of hospital stay, readmission, and discharge to a facility, as this information will likely have implications on their future quality of life and willingness to undergo surgery. For example, many patients with terminal disease prefer to die at home, as prior research has shown that in-hospital death among cancer patients is associated with greater physical and emotional distress and worse quality of life.[5, 25] However, this end of life goal may not be met if surgical intervention is pursued, as our data show that such intervention places patients at greater risk for prolonged hospital stay, readmission, discharge to a facility and death within 30 days.

Our findings that DMa patients who did not experience a single complication nevertheless experienced an increased risk of prolonged length of hospital stay, readmission, discharge to a facility and death compared to non-DMa matched controls also has important implications for this patient population. It suggests that the diagnosis of DMa itself is a predictor of poorer outcomes following surgical intervention. This may be due to many factors including greater patient frailty, weight loss and malnutrition among DMa patients, as these are known factors of adverse postoperative outcomes among cancer patients.[11, 26, 27] However, irrespective of whether DMa is causally related to worse post-surgical outcomes or simply associated, the finding of higher rates of prolonged hospital stay, readmission, and discharge to a facility in the absence of a post-surgical complication undermines an important rationale for performing surgery in this patient population, namely that patients who are successfully palliated by surgery derive significant benefits. In fact, our data suggest that even patients whose surgery is uncomplicated experience adverse outcomes.

Despite such findings, we understand that there are instances when surgical management will remain indicated and potentially life-saving for DMa patients. In such times, it is important that surgeons understand all factors that further increase DMa patients surgical risk. Our findings support prior literature identifying predictors of acute morbidity and mortality in DMa patients to include increased age, male gender, poor functional status, preoperative dyspnea and sepsis, ventilator dependence, lower albumin, emergency operations, and multivisceral resections.[10, 11] We found similar predictors of prolonged hospitalizations, disposition to facilities other than home, and hospital readmissions among DMa patients. These findings highlight the need for surgeons and other members of the healthcare team to clearly engage in goals of care and end of life discussions with DMa patients since patients may experience adverse outcomes beyond that of traditional acute surgical morbidity, and these outcomes may impact their performance status, independence, as well as quality of life.

There are limitations of our study. Most importantly, we were not able to compare differences in outcomes between operative and nonoperative management since ACS-NSQIP only captures data on surgical patients. Although our findings demonstrate the significant risks that DMa patients harbor for acute morbidity and mortality as well as prolonged hospital stay, readmission, and discharge to a facility following surgery, it is inappropriate and premature at this time to extrapolate these data to DMa patients who do not undergo surgery for similar surgical conditions. This is an important subject for future investigation. Additionally, despite the robust nature of ACS-NSQIP data,[12] patient outcomes greater than 30 days postoperatively are not available. It is conceivable that DMa patients are more likely to experience the outcomes of interest more quickly than non-DMa patients, thereby biasing our results given the 30-day ascertainment period. Furthermore, cause of death data is not available in ACS-NSQIP data and, thusly, it is unclear if the increased risk of 30-day mortality among DMa patients is secondary to their terminal cancer diagnosis or the surgical intervention performed. Regardless of cause of death, we contend, along with other cancer researchers, that invasive treatments including chemotherapy and surgery near the end of life is indicative of poor patient selection and arguably less than optimal quality of care.[1–3] Lastly, we did not have information regarding the goals of surgical intervention (i.e. palliation vs. prolong life). Future research is warranted to address these limitations, including evaluating postoperative outcomes and quality of life measurements for DMa patients who undergo surgical versus medical management of a “surgical” condition, such as bowel obstruction.

In conclusion, we demonstrate that DMa patients who underwent surgical intervention experienced increased rates of prolonged length of hospital stay, readmissions, minor and major complications, discharges to facilities other than home, and death. These adverse outcomes were demonstrated across the spectrum of surgical conditions. Such findings emphasize the need for physicians, surgeons, and other members of the healthcare team to engage in goals of care and end of life discussions with DMa patients prior to pursuing surgical intervention since patients may experience adverse outcomes beyond that of traditional acute surgical morbidity, and these outcomes may impact their performance status and independence.
